# Study of clopidogrel and clonidine interactions for cardiovascular formulations: progress from DFT modeling

**DOI:** 10.1039/d4na00776j

**Published:** 2025-02-21

**Authors:** B. Ocampo Cárdenas, G. Román, E. Noseda Grau, S. Simonetti

**Affiliations:** a Universidad del Quindío Carrera 15 Con Calle 12 Norte Armenia Quindío Colombia BOCAMPOC@uqvirtual.edu.co; b Universidad Tecnológica Nacional, Facultad Regional Bahía Blanca 11 de Abril 461 B8000LMI Bahía Blanca Buenos Aires Argentina; c Instituto de Física del Sur (IFISUR), Departamento de Física, Universidad Nacional del Sur (UNS), CONICET Av. L. N. Alem 1253 B8000CPB – Bahía Blanca Argentina

## Abstract

The drugs clopidogrel and clonidine are frequently used to treat cardiovascular diseases, which are the leading cause of mortality worldwide. Since these medications are frequently taken in combination, it is crucial to examine their molecular interactions. Therefore, herein, the bandgap energy, chemical potential, chemical hardness and softness parameters were calculated using a density functional theory (DFT)-based method. In addition, infrared (IR) spectrum, natural bond orbital (NBO), molecular electrostatic potential (MEP), electron localization function (ELF) and total density of states (TDOS) plots complemented the analysis. Clonidine exhibited greater sensitivity to electrophilic attack, while the electronic affinity of clopidogrel was slightly higher. According to the MEP map, negative charge density was located on the oxygen atoms of clopidogrel, and the positive charge was located on the nitrogen atoms of clonidine. Notably, both the drugs exhibited similar reactivity in water. Clopidogrel was less reactive than clonidine, and the interaction between the molecules occurred *via* physisorption, which was in agreement with the TDOS plot. NBO analysis revealed a low charge variation, in accordance with the physical adsorption-like bonding between the drugs. The lowest energy for the clopidogrel–clonidine interaction was attained *via* the formation of four H bonds, as indicated by a significant intensive peak at 3360 cm^−1^ in the IR spectrum. Hydrogen bonds played a crucial role in the controlled drug delivery application as it allowed moderate and reversible drug adsorption, facilitating drug release in the biological environment. IR spectra also supported the absence of degradation or chemical reaction between the drugs, confirming the preservation of the individual active pharmaceutical ingredient.

## Abstract 2

The drugs clopidogrel and clonidine are frequently used to treat cardiovascular diseases, which are the leading cause of mortality worldwide.

## Graphical abstract

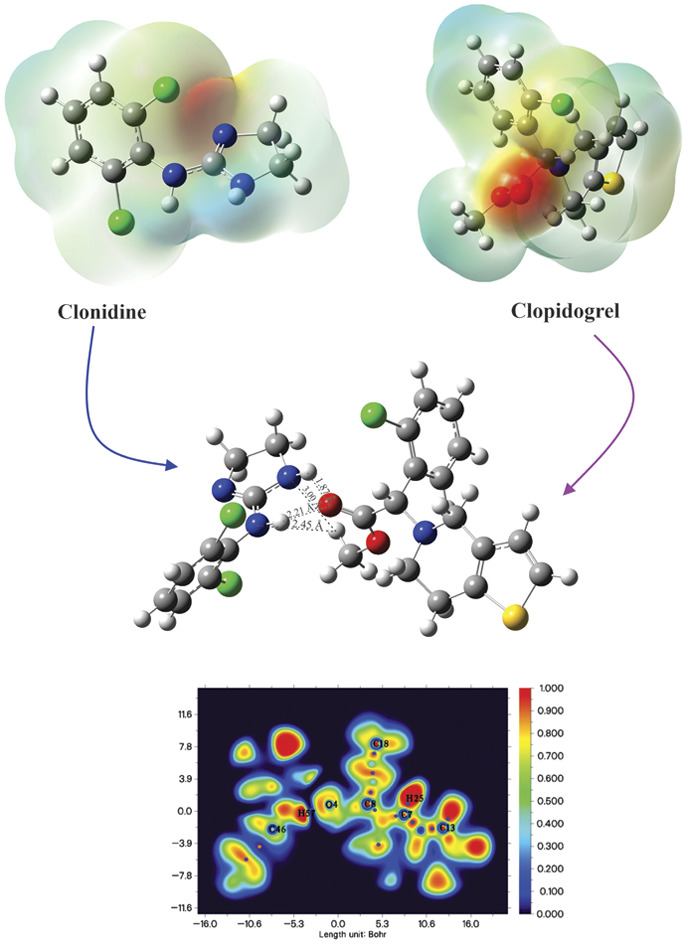

## 1. Introduction

Cardiovascular diseases (CVDs) are the leading cause of mortality globally, registering 20.5 million CVD-related deaths in 2021.^1^ Cardiovascular medicines, such as clopidogrel and clonidine, are critical for the prevention and management of CVDs for long-term treatment of patients.

**Fig. 1:**
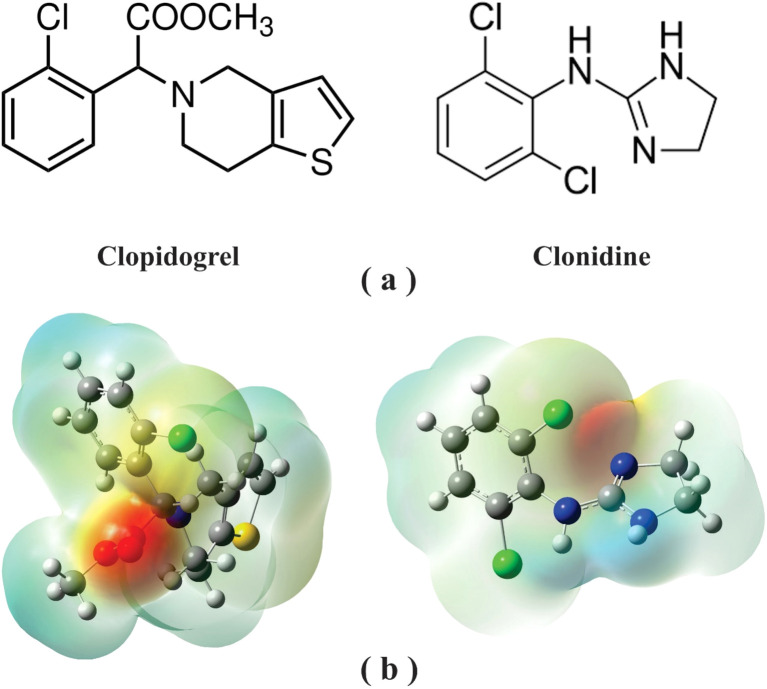
(a) Chemical structure and (b) molecular electrostatic potential (MEP) of clopidogrel and clonidine drugs.

Clopidogrel (Fig. 1(a)) is an analogue of ticlopidine.^2^ Its chemical name is methylalpha-(2-chlorophenyl)-6,7-dihydrothieno[3,2-*c*]pyridine-5(4*H*)acetate. Clopidogrel is a prodrug with pharmacological activity since antiplatelet activity is dependent on its hepatic transformation into a thiol metabolite through oxidation and hydrolysis. In the coagulation mechanism, platelets adhere to the sub-endothelium that is exposed after tissue injury. After this first phase of adhesion, platelets combine with other platelets in the process called aggregation, forming the thrombus. The platelet receptor, GPIIb–IIIa,^3^ actively participates in both the processes. Clopidogrel blocks^4^ the platelet adenosine diphosphate (ADP) receptor.^5^ In this way, the binding of fibrinogen to the GPIIb–IIIa glycoprotein receptor^6^ is inhibited, preventing the continuity of the aggregation cascade. Clopidogrel acts by reducing the fibrinogen–GPIIb–IIIa binding without altering this complex.^7^ The platelet modification caused by the clopidogrel active metabolite is irreversible.^8^ In 2022, clopidogrel was ranked 47^th^ in the top 200 prescribed medications in the United States.^9^ Owing to the importance of clopidogrel in the pharmaceutical industry, many studies have been reported recently. Forced degradation studies that investigate the intrinsic stability of clopidogrel under various stress conditions have attracted the interest of the scientific community and pharmaceutical industries.^10,11^ A study shows that patients at extreme CVD risk have more genetic resistance to clopidogrel.^12^ These patients have more coronary events; therefore, the genetic resistance to individualized antiplatelet treatment should be studied. Raman spectra simulation was performed to study the interaction of clopidogrel metabolite with the P2Y12 receptor.^13^ The theoretical data were compared with the experimental SERS results. The prospects of obtaining results for pathologies based on platelet conformations during cardiovascular diseases have been successfully demonstrated.^13^

Clonidine (Fig. 1(a)), the imidazole alpha-2-adrenergic agonist, was introduced into clinical practice first as a nasal decongestant, and it displays systemic effects, such as arterial hypotension, bradycardia and sedation.^14^ Once the hypotensive effect of clonidine in the central and peripheral nervous systems was demonstrated, it began to be prescribed to control high blood pressure. In recent decades, the advantages of clonidine have been recognized and it has been used for anesthesia in cardiac surgeries. Among other benefits, the reduction of opioid administration in the intra- and post-operative periods was attributed to clonidine, which allows rapid tracheal extubation and short periods of mechanical ventilation. The cardiovascular action of clonidine is well known. Its vasodilatory activity is due to the peripheral and central mechanisms that lead to a reduction in blood pressure.^15–17^ In 2022, clonidine was ranked 71^st^ in the top 200 prescribed medications in the United States.^9^ Due to the importance of clonidine in the pharmaceutical industry, many studies have been reported from past years to the present. Experimental and theoretical approaches were combined to investigate the thermodynamic and kinetic characteristics of clonidine oxidation in remedial studies.^18^ On the other hand, structural properties and FTIR-Raman spectra of the clonidine hydrochloride and their dimeric species were determined.^19^ The knowledge of the structures and vibrational properties of these anti-hypertensive agents is essential for their quick identification for the synthesis of new derivatives with improved pharmacologic properties and to know and explain their behavior and mode of action.^19^ The effects of clonidine on the cardiovascular, renal, and inflammatory responses to experimental bacteremia were also studied.^20^ High clinical doses of clonidine attenuated the sepsis-related increase in the heart rate and cardiac output, with negligible effect on arterial pressure. It also induced water diuresis, reduced body temperature, and had an anti-inflammatory action. Low-dose clonidine had similar but less pronounced effects, except that it induces moderate vasodilatation and increases cardiac output. A study was performed to reveal the benefit of clonidine as an adjuvant therapy to bupivacaine.^21^ Low doses of clonidine along with bupivacaine show no side effects and well-maintained systolic blood pressure with analgesia for longer periods of time.

In recent years, there has been an increased interest in artificial intelligence and computational methods.^22^ The DFT-based studies for the interaction of different molecules with various substrates have been published recently.^23–33^ Using DFT calculations, the adsorptions of SO_2_, SO_3_ and O_3_ gas molecules on the MoS_2_ monolayers were studied in terms of adsorption energy, charge transfer, band structures, and charge density differences.^23^ Various adsorption geometries and sites were considered in detail for the adsorption behaviour of SO_*x*_ molecules on the pristine and N-doped ZnO nanoparticles using DFT calculations.^24^ Theoretical investigation of CO and NO gas molecules shows shorter adsorption distances and higher adsorption energy for the Al-doped and Si-doped monolayers than the P-doped and pristine ones.^25^ The charge density difference calculations show the charge accumulation between the interacting atoms, suggesting the formation of covalent bonds, as evidenced by the projected density of states of the interacting atoms.^25^ The DFT study of the adsorption of O_3_ and NO_2_ molecules on ZnO nanoparticles provides a theoretical basis, giving rise to the design of innovative and highly efficient sensor devices.^26^ Theoretical findings show that the TiO_2_/stanene heterostructure holds great promise for fabricating novel and highly sensitive NO_2_ and O_3_ gas sensors.^28^ The interaction of gas molecules (CO, NO, N_2_O and NH_3_) with Pd-decorated stanene nanosheets was studied using DFT calculations.^29^ The considerable electron orbital overlaps between the gas molecules and Pd-embedded stanene confirm that strong chemisorption occurs, showing great potential as a promising candidate for gas sensing.^29^

Cardiac diseases are one of the principal causes of death worldwide. In these situations, patients receive multifaceted pharmacological pills that result in a low adherence rate. The combination of drugs has been shown in clinical studies to increase the anti-hypertensive efficacy of both agents compared to either agent alone.^34^ Consequently, a formulation containing clonidine and clopidogrel drugs for cardiovascular treatment could improve the treatment outcomes in patients. In this context, it is crucial to study the drug properties and the drug–drug interactions in their chemical environment. The objective of this research is to obtain a more complete understanding of the physical phenomena that occurs in this multi-drug system. This work emphasizes the role of computational chemistry as a clean technique in analyzing drug–drug interactions, paving the way for future experimental research in this field.

## 2. Theoretical method

The isolated molecules and configurations have been modeled with GaussView05,^35^ and all calculations were done using the Gaussian 09 package.^36^ Full geometry optimizations were performed using DFT with a correlation functional of Lee, Yang, Parr (B3LYP) level using the 6-31G basis set.^37–39^ Dispersion effects were incorporated by the correction-based Grimme's (D3) approach.^40^ We highlight the efficacy of the functional and basis set used in this study. B3LYP is a commonly used functional in various systems, while the 6-31G basis set is suitable for general calculations on medium to huge molecules and it is an optimal level of theory to deliver reliable results at minimum cost.^40–43^ Real biological environments are mimicked by optimizing all the studied structures in the water solvent. Solvent effects are incorporated in calculations using the polarizable continuum model,^44^ which is a continuum solvent model already well-developed and widely used in treating solvent effects on molecular properties^45^ due to the reliability of the model.^46^

There is a strong relationship between the quantum chemical parameters linked to the electronic structure and the chemical behavior of molecules during interactions.^47,48^ The chemical parameters can be calculated when molecular orbitals, such as the HOMO (Highest Occupied Molecular Orbital) and the LUMO (Lowest Occupied Molecular Orbital), are available.^49^ From the frontier orbitals, we can determine the energy band gap, defined as,

1 *E*_g_ = (*E*_LUMO_ − *E*_HOMO_)

which indicates the activity of the molecule in chemical reactions. From the band gap, we can define a series of parameters.

We can describe the hardness “*η*” as,

2 *η* = (*E*_LUMO_ − *E*_HOMO_)/2

Hardness is defined as the measure of the resistance to deformation of the chemical structure in an external electric field. The increase in hardness implies increased stability. On the contrary, if the gap is small, it can be described by the global softness (*S*),

3 *S* = 1/(2*η*)

which is the reciprocal of hardness.

Another descriptor is the electronic chemical potential (*μ*),

4 *μ* = (*E*_HOMO_ + *E*_LUMO_)/2

which is associated with the ability of the molecule to exchange electron density.

Different interaction mechanisms between the drugs are involved in the systems; therefore, we can define the electrophilicity (*ω*) as,

5 *ω* = *μ*^2^/2*η*

which measures the change in the energy of an electrophile species when it becomes saturated with electrons.

The different clopidogrel–clonidine configurations were optimized, and we have calculated the adsorption energy (Δ*E*) as

6 Δ*E* = *E*_clopidogrel–clonidine_ − (*E*_clopidogrel_ + *E*_clonidine_)

The positive value of Δ*E* indicates repulsion between the drugs, while its negative value indicates attraction.

## 3. Results and discussion

### 3.1. Theoretical parameters

Firstly, we analyze the differences in reactivity of all the species: cationic, neutral and anionic species of clopidogrel and clonidine drugs. We defined the reactivity of the molecule as its activity or capacity to take part in a reaction or interaction with another species. Table 1 shows the calculated quantum chemical parameters. The HOMO energy (*E*_HOMO_) is associated with the electron-donating capacity, while the LUMO energy (*E*_LUMO_) is associated with the ability to accept electrons. Thus, the frontier orbitals are very important in defining the interaction between the molecules. The *E*_HOMO_ value (see Table 1) describes the sensitivity of the species to any electrophilic attack in the region where the HOMO is located. Analyzing the three species of clonidine, we can observe that clonidine1 presents a larger *E*_HOMO_ value (−7.483) compared to clonidine0 (−5.442) and clonidine−1 (−5.360). This result indicates that clonidine1 has greater sensitivity to an electrophilic attack than the other species. On the other hand, clopidogrel1 presents higher *E*_HOMO_ (−6.884) compared to the other species whose values are −6.014 eV (clopidogrel0) and −5.442 eV (clopidogrel−1). Note that the energies are similar for all the species in this case. A high value of *E*_HOMO_ represents the tendency of the molecule to donate electrons to an acceptor species, which may be promising for the interaction between these donor and acceptor species.

**Table 1:** Values of the highest occupied molecular orbital energies (*E*_HOMO_) and lowest unoccupied molecular orbital energies (*E*_LUMO_), energy gap (*E*_g_), global hardness (*η*), chemical potential (*μ*), electrophilicity (*ω*) index and softness (*S*) (all in eV)

Species^a^	*E*_HOMO_	*E*_LUMO_	*E*_g_ b	*η* c	*μ* d	*ω* e	*S* f
Clonidine1	−7.483	−1.306	6.150	3.075	−4.381	240.327	3.129
Clonidine0	−5.442	−0.898	4.544	2.286	−3.184	325.335	2.231
Clonidine−1	−5.360	−0.517	4.843	2.422	−2.939	306.287	1.796
Clopidogrel1	−6.884	−1.524	5.360	2.667	−4.218	276.736	3.320
Clopidogrel0	−6.014	−0.898	5.116	2.558	−3.456	289.960	2.340
Clopidogrel−1	−5.442	−0.626	4.816	2.422	−3.048	307.239	1.905

a Clonidine/clopidogrel 1 = cationic species, (+1) charged, clonidine0/clopidogrel0 = neutral species and clonidine−1/clopidogrel−1 = anionic species, (−1) charged.

b Eqn (1).

c Eqn (2).

d Eqn (4).

e Eqn (5).

f Eqn (3).

The magnitude of the *E*_LUMO_ (see Table 1) indicates the ability of the molecule to accept electrons. If the molecule presents high *E*_LUMO_, the probability of the species accepting electrons decreases. In the case of the clonidine drug, clonidine1 (−1.306) has a larger value than clonidine0 (−0.899) and clonidine−1 (−0.517), while for clopidogrel, a higher value is presented in clopidogrel1 (−1.524) compared to clopidogrel0 (−0.898) and clopidogrel−1 (−0.626), respectively. Therefore, clonidine1 has a greater capacity to donate electrons than the other species, while clopidogrel1 has a greater affinity to acquire electrons. It is important to note that, in all the cases, the species present the same *E*_HOMO_/*E*_LUMO_ ratio; thus, these parameters cannot indicate a marked trend by themselves and it is necessary to analyze other parameters.

A relatively higher bandgap (*E*_g_) value clearly indicates that the molecule is more stable, that is, less reactive. In the case of clonidine (see Table 1), the most stable species is clonidine1 (6.150) and the most reactive is clonidine0 (4.544). In the case of clopidogrel, the most stable is clopidogrel1 (5.360); nevertheless, for all the configurations, the *E*_g_ values are not significantly different. Note that clopidogrel0 has a bandgap energy slightly higher than clonidine0, and the type of interaction that will occur between the molecules is influenced by this parameter.

The chemical potential (*μ*) is related to the electronegativity. This parameter is associated with the changes in the electron density, that is, the possibility of electrons flowing from a region of high potential or low electronegativity to another region of low potential or high electronegativity. In our study, clonidine1 (−4.381) and clopidogrel1 (−4.218) were the most electro-attractive (see Table 1), being the species that present the lower chemical potential (greater electronegativity) compared with the other species. In the case of clonidine0 (−3.184), clonidine−1 (−2.939), clopidogrel0 (−3.456) and clopidogrel−1 (−3.048), these species have higher chemical potential and lower electronegativity.

Chemical hardness (*η*) is a direct measure of the band gap; a large band gap implies a high chemical hardness value, that is, the species is lesser reactive or liable, or both, to receive electrons and to donate them. In our study, it is clearly observed that clonidine1 (3.075) has an *η* value significantly higher than the other species (see Table 1). This indicates that clonidine1 is less reactive than the other species; that is, it is more chemically stable. For comparison, the rest of the species have similar reactivity (clonidine0 (2.286), clonidine−1 (2.422), clopidogrel1 (2.667), clopidogrel0 (2.558) and clopidogrel−1 (2.422)). On the other hand, the chemical softness (*S*) parameter is the reciprocal of the hardness. As expected, the most chemically reactive is clopidogrel1 (3.320). Its *S* value is very close to clonidine1 (3.129). Then, all the other species have similar or closer *S* values (see Table 1). Note that the clonidine−1 and clopidogrel−1 species have an *S* value slightly lower than the other species.

The electrophilicity index (*ω*) is used to measure the decrease in the energy produced by the maximum flow of electrons between the HOMO (donor) and the LUMO (acceptor). A high value of this parameter indicates that the molecule will be more resistant to change in its electronic distribution. Clonidine1 presents the lowest *ω* value (240.327) (see Table 1), while clonidine0 presents a slightly larger *ω* value (325.335), very similar to clonidine−1 (306.287) and clopidogrel−1 (307.239). The electrophilicity depends proportionally on the electronegativity and inversely on the chemical resistance or stability. If the species has both high electronegativity and chemical resistance, it would have low electrophilicity. If we analyze, for example, the case of clonidine1, which is the most electro-attractive, it presents the greatest electronegativity and chemical resistance and the lowest electrophilicity. The rest of the species have very similar electronegativity and lower electronegativity than clonidine1. They also present lower chemical resistance, with the exception of clopidogrel1, which has similar chemical stability to clonidine1. In general, when the chemical resistance is low, the species is more reactive and presents a high electrophilicity value. It is important to highlight the cases of clonidine1 (which presents high electronegativity and high chemical resistance) and clopidogrel1 (which presents low electronegativity and high chemical resistance), and in both cases, they have very different values of electrophilicity. We can conclude that electronegativity is the parameter that determines the behavior of the species. The electronegativity/chemical resistance ratio indicates which species receive the electrons (electrophilic capacity) or donate electrons (nucleophilic capacity). In our study, the cationic clonidine has nucleophilic capacity (*ω* = 240.327), while the anionic (*ω* = 306.287) and neutral (*ω* = 325.335) species present electrophilic capacity. In the case of clopidogrel, this drug has an electrophilic capacity between 276.736 and 307.239.

### 3.2. Geometry optimization

**Fig. 2:**
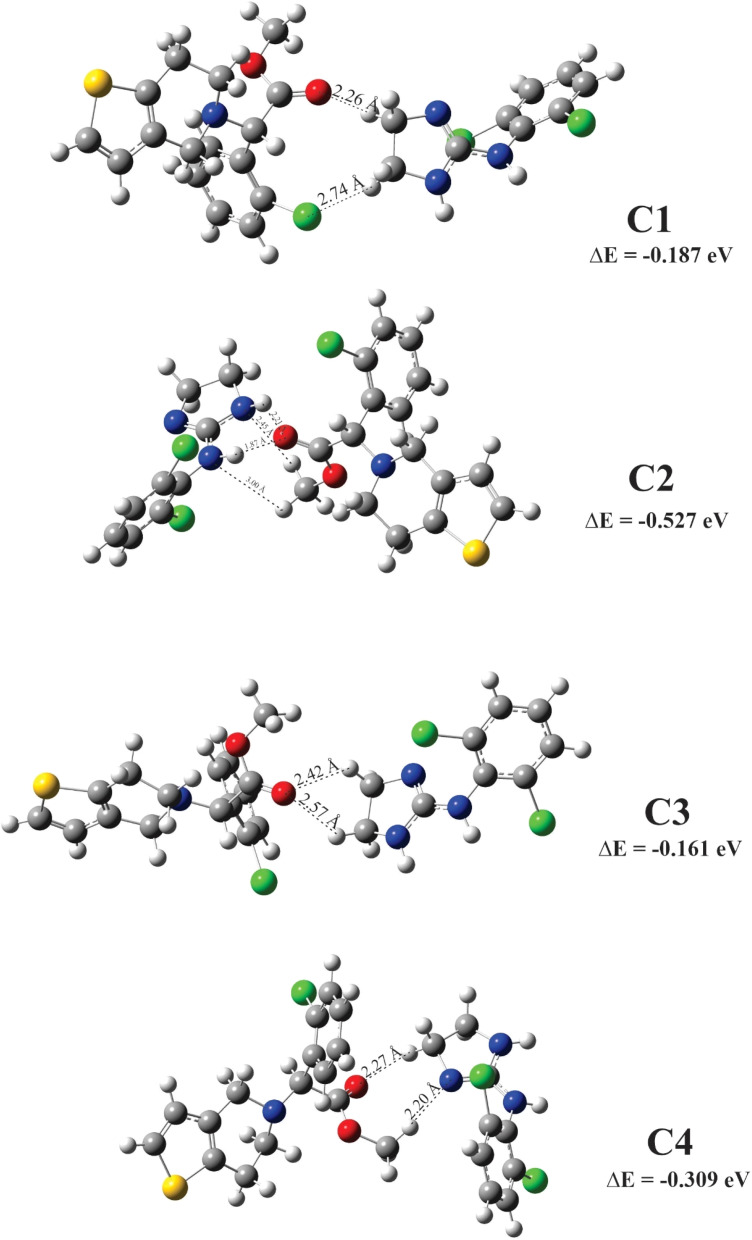
Configurations on minimum energy for clopidogrel–clonidine interaction.

**Fig. 3:**
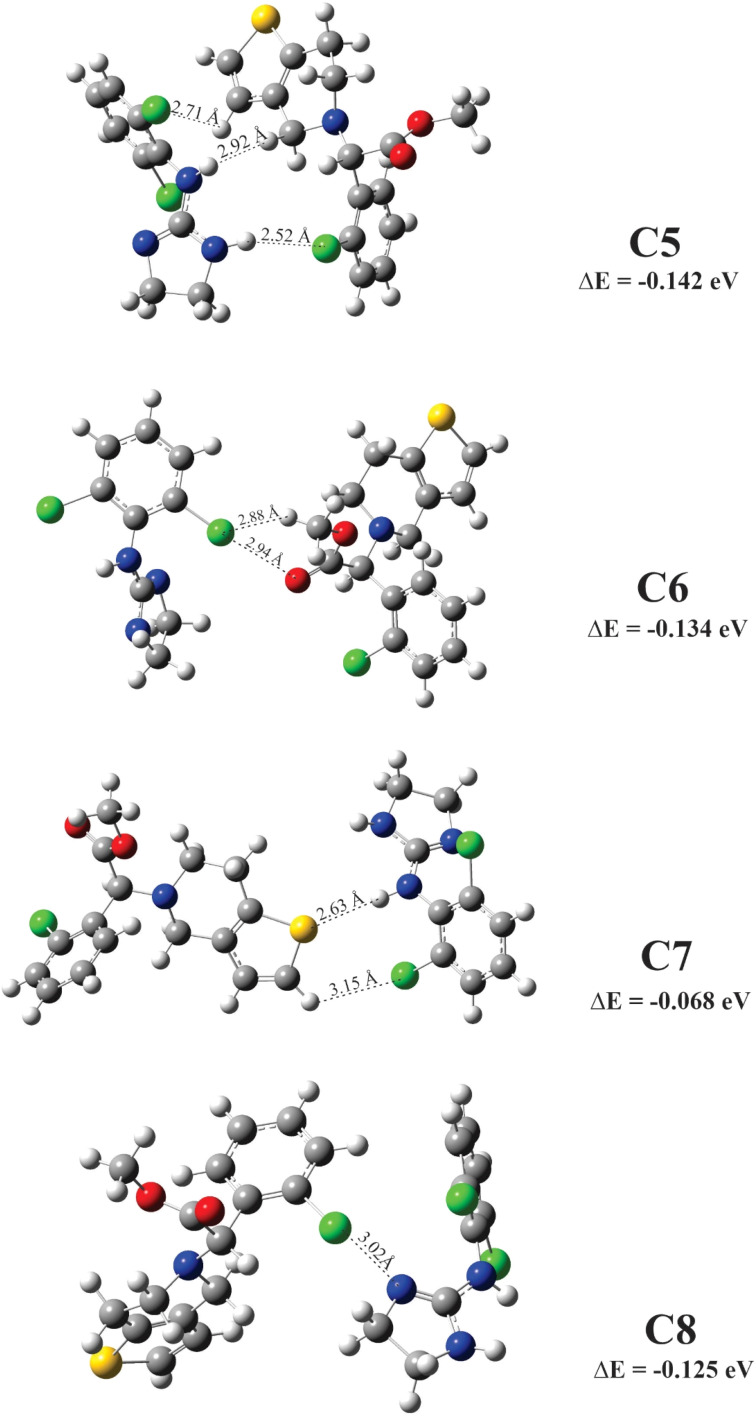
Configurations on minimum energy for clopidogrel–clonidine interaction.

**Fig. 4:**
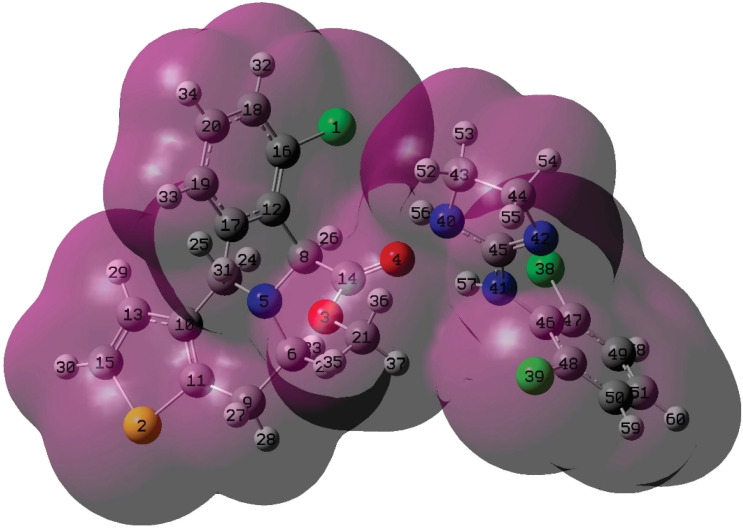
Electron density from total SCF density for C2 configuration. Numbering for atoms is showed.

The molecular electrostatic potential (MEP) for clopidogrel and clonidine molecules is shown in Fig. 1(b). The negative charge density (red color) is concentrated on the oxygen atoms of clopidogrel, while the positive charge density (blue color) is located on the nitrogen atoms of clonidine. These charge concentrations predict a high probability of interaction between the regions with opposite charge densities. Analyzing the complex structure of clonidine, this molecule presents an imidazole ring, which contains two characteristic nitrogens belonging to a ring of five atoms, an amino group, and a benzene ring containing two chlorine atoms in *ortho* positions. In the case of clopidogrel, among its most characteristic functional groups, it presents a carboxy-ester group, a benzene ring containing a chlorine atom, and a thiophene ring joined to an adjacent pyridine ring by *ortho* fusion. This structural complexity generates particular charge densities and concentrations of active sites, positioning both molecules in their most favorable geometries for the interaction. Fig. 2 and 3 show the optimized clopidogrel–clonidine configurations. As shown in Table 2, all the systems are stable because the obtained energies are negative. All the configurations present energies of small absolute values (see Table 2), typical of systems formed through physical interactions. Typical binding energy of physisorption is about 10–600 meV, while chemisorption usually forms a bonding with energy of 1–10 eV. In general, physisorption involves low-energy interactions.^50^ The most favorable configuration is C2, which has the lowest energy of −0.527 eV (see Table 2). Fig. 4 shows the total electron density plot for the C2 configuration. In the C2 system, the opposite charge densities (Fig. 1) favorably interact without significant steric impediment, and thus, the interaction between the drugs is produced with the lowest energy. This occurs when both molecules are positioned, exposing their most active zones and reaching the C2 geometry with lesser steric hindrance. The interaction between the drugs is produced by two O–H bonds and two N–H bonds, *i.e.*, through four H-bonding interactions (see Fig. 2). The oxygen of the carbonyl group of clopidogrel forms two hydrogen bonds with the hydrogens of clonidine at O–H distances of 1.87 Å and 2.21 Å, respectively; while the amino nitrogen of the imidazole ring of clonidine with a carbonyl group (O=C) of clopidogrel forms two hydrogen bonds at N–H distances of 2.45 Å and 3 Å, respectively. Similar bonds and energies are reported in the literature.^51^ These H-bonding interactions contribute to determining the optimal geometry for the C2 configuration being consistent with the obtained adsorption energy. Hydrogen bonds play a crucial role in determining the specificity of molecule binding, which is important in stabilization. Furthermore, during administration, the impact of hydrogen bonds on passive diffusion across cell membranes is important. Then, new hydrogen bonds will easily form with the polar structure of water and dissolving drug substances required by the organism, which is necessary for biochemical reactions. H-bonding interactions are ideal for controlled drug delivery applications as it allows moderate and reversible drug adsorption, facilitating its release in the biological environment.

**Table 2:** Energies for the clopidogrel–clonidine configurations

Configurations^a^	Δ*E*^b^ (eV)
C1	−0.187
C2	−0.527
C3	−0.161
C4	−0.309
C5	−0.142
C6	−0.134
C7	−0.068
C8	−0.125
*E*_clopidogrel_ = −231.039 eV	
*E*_clonidine_ = −143.176 eV	

a Fig. 2 and 3.

b Eqn (6).

### 3.3. Infrared spectrometry (IR)

A widely used tool for probing the internal structure of an organic molecule is the infrared (IR) spectrometry technique. IR spectrum is the fingerprint for any compound as it represents different bonds, functional groups and vibrations. IR spectrophotometry allows us to identify shifts in the vibrational frequencies of the functional groups involved in the covalent and non-covalent interactions.^52,53^ Chemical interactions result in the breaking and formation of new bonds, changing the chemical nature of a compound. Any change in the IR spectra of the compounds could predict changes in their chemical structure and indicate drug degradation or chemical bonding between the drugs. Therefore, IR spectroscopy is also performed to determine the compatibility between the drugs.

**Fig. 5:**
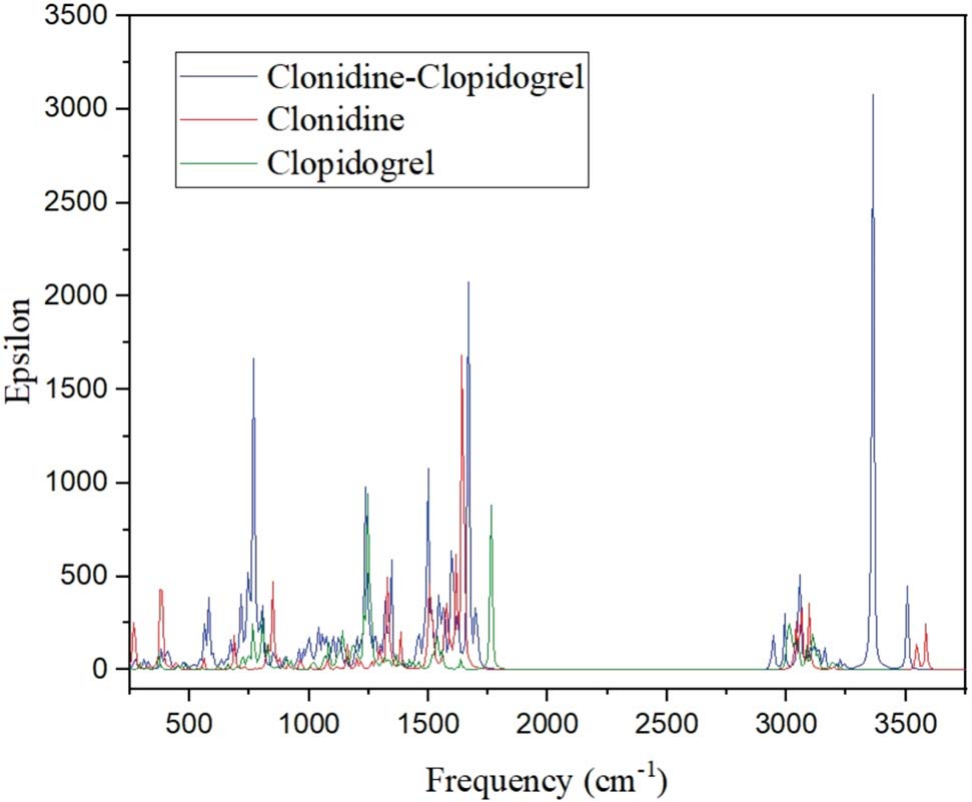
IR spectra of isolated clopidogrel, isolated clonidine and clopidogrel–clonidine interaction (C2).

A detailed study of the spectra reveals the presence of characteristic peaks associated with certain functional groups in the isolated molecules, as well as bonds that could be formed after the interactions (C2).^54^ The spectra in Fig. 5 show the presence of bands attributed to both drugs. The IR spectrum shows a band due to the C=O stretching vibrations at 1699 cm^−1^ corresponding to clopidogrel. The band due to aromatic C–H stretching vibrations is represented at 3160 cm^−1^. The bands associated with C–O stretching appear at 1068, 1153 and 1194 cm^−1^. The IR spectrum includes peaks at 3005 cm^−1^, which can be attributed to the stretching vibrations of the bonded N–H of clonidine. The bands with different intensities observed at 1667 cm^−1^ and 1245 cm^−1^ are assigned to the C–N stretching modes, while the band at 445 cm^−1^ is assigned to the C–N–C deformation. The group of bands between 3097 and 3105 cm^−1^ are assigned to the C–H stretching modes. The corresponding in-plane deformations modes are assigned to the IR bands at 1438, 1199 and 1157 cm^−1^ while the bands at 976, 900 and 768 cm^−1^ are assigned to the corresponding out-of-plane deformations for all the species. The very weak band at 266 cm^−1^ and 222 cm^−1^ could be assigned to the stretching modes related to the C–H⋯Cl modes. The asymmetric and symmetric stretching modes of CH_2_ are assigned between 3600 and 2944 cm^−1^. The IR bands at 1499, 1484 and 1457 cm^−1^ are assigned to the scissoring modes in agreement with compounds containing the CH_2_ group, while the medium IR bands at 1342 and 1293 cm^−1^ are assigned to the wagging modes. The expected rocking modes are assigned to the weak IR band at 1199 cm^−1^. The twisting modes are associated with the weak band and a shoulder in IR at 1026 and 805 cm^−1^, respectively. The C=C stretching modes of the phenyl ring were assigned to the bands at 1593, 1568, 1299 and 1073 cm^−1^. The C–Cl stretching modes are assigned to the IR bands at 411 and 398 cm^−1^. However, the corresponding out-of-plane deformation modes appear at 521 and 505 cm^−1^ in the IR. Two weak bands in the IR spectrum at 1096 and 686 cm^−1^ are assigned to the in-plane deformation modes of the phenyl ring (bendings). Signals appearing in the IR spectrum at 1123 cm^−1^ and 1100 cm^−1^ are assigned to the in-plane deformation of the phenyl rings. For the imidazole ring, these bands appear at 867 and 686 cm^−1^ in the IR. Moreover, the torsional ring modes for the phenyl ring are assigned to the IR bands at 695 and 480 cm^−1^. The peaks of the vibrational modes around 3131 cm^−1^ and near 3505 cm^−1^ correspond to the vibrations of the O–H and the H–N bonds, respectively.^55^ In the case of the clonidine–clopidogrel system (C2), the peaks appear with very significant intensity in relation to the isolated molecule peaks, and, especially, the peak at 3360 cm^−1^ could be associated with the formed H-bonds. H-bonds are formed by the approach of the molecules producing the characteristic vibrational mode of these functional groups, as can be seen in the IR spectrum (Fig. 5). This finding corroborates the O–H and N–H bonds formed during the C2 interaction. On the other hand, the appearance of the same characteristic peaks of isolated clonidine and clopidogrel in the C2 spectrum, without any changes in their position, indicates the absence of the degradation of drugs or chemical bonds between the drugs.

### 3.4. Total density of states (TDOS)

**Fig. 6:**
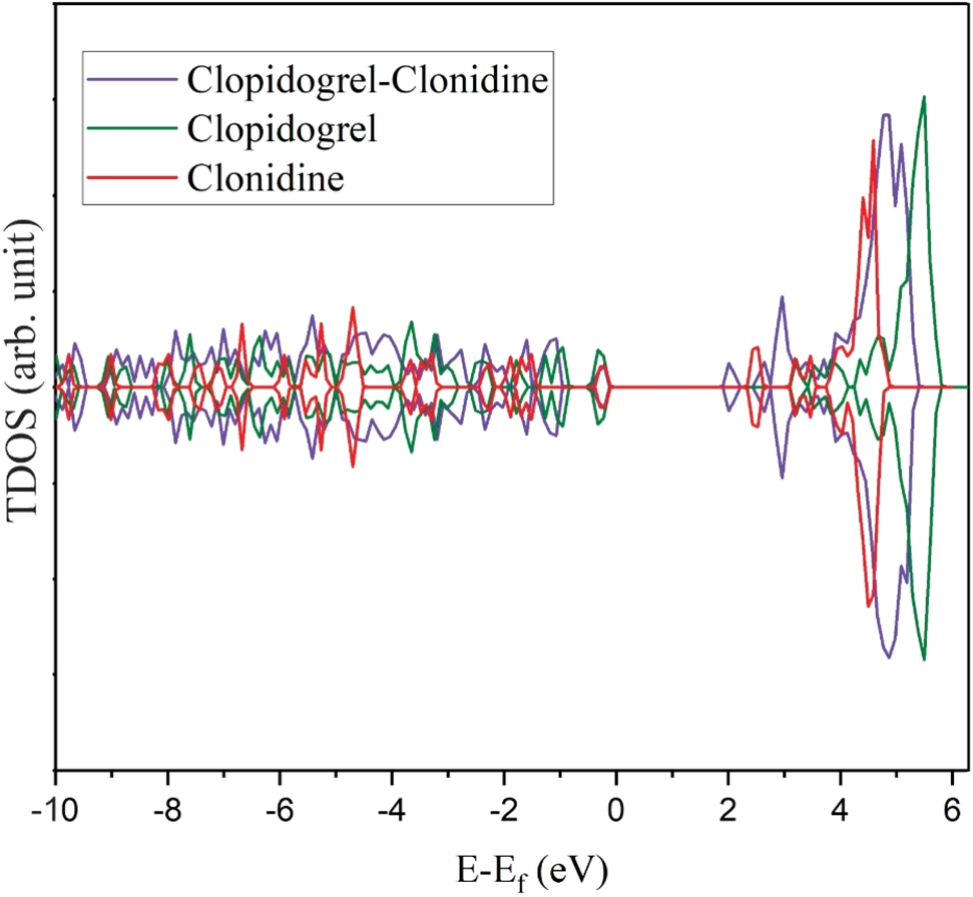
Total density of states (TDOS) for clopidogrel–clonidine (C2), isolated clopidogrel and isolated clonidine.

The total densities of states (TDOS) of each isolated molecule and the C2 configuration are presented in Fig. 6. Superposition can be seen between the molecule bands. It can be observed that the clopidogrel molecule has a larger bandgap than clonidine, corroborating the *E*_g_ value presented in Table 1. The clopidogrel–clonidine system (C2) is more stable than the isolated molecules because their molecular states are stabilized with lower energy values after the interaction. At the time of the interaction between the molecules, new states are generated in the area close to the Fermi level. However, the size of the remanent gap plus the absence of states precisely at the Fermi level coincides with the previous finding, that is, the interaction between the drugs is produced by physical adsorption.

### 3.5. Atomic charge distribution

In order to analyze the interaction in detail, we have calculated the atomic charge distributions (natural bonding orbital-NBO)^56^ for the C2 configuration and the isolated drugs (Table 3). As shown, the charge exchanges are small. It can be associated with the physical adsorption between the drugs. The atom that is the most affected in clopidogrel is O4, which has a charge variation of −0.041, belonging to the carbonyl group that participates in the two H-bond formations previously described. This is due to the proximity of H56 and H57 of clonidine, which promotes a redistribution of charges, electro-negativizing the oxygen. On the other hand, the main redistribution on clonidine occurs on N40 and N41, which present charge modifications of −0.015 and −0.018, respectively. This is in agreement with the formation of two N–H bonds in C2, as previously described. It is important to mention that an organic molecule undergoes charge redistributions to stabilize itself in the new electrochemical environment generated by the approach of another organic molecule. This is much more accentuated in the regions close to the zone of interaction. Distant to this zone, the molecules present an electronic rearrangement that stabilizes them without major changes.

**Table 3:** NBO charge for isolated clopidogrel, isolated clonidine and clopidogrel–clonidine interactions (C2)

Atom^a^	No.	Clopidogrel–clonidine	Clopidogrel	Clonidine	Δcharge
Cl	1	−0.053	−0.057	—	0.004
S	2	0.330	0.328	—	0.002
O	3	−0.472	−0.491	—	0.019
O	4	**−0.571**	**−0.530**	—	**−0.041**
N	5	−0.498	−0.497	—	−0.001
C	6	−0.266	−0.266	—	—
C	7	−0.277	−0.277	—	—
C	8	−0.157	−0.160	—	0.003
C	9	−0.496	−0.496	—	—
C	10	−0.104	−0.104	—	—
C	11	−0.172	−0.172	—	—
C	12	−0.078	−0.076	—	−0.002
C	13	−0.277	−0.277	—	—
C	14	**0.776**	**0.739**	—	**0.037**
C	15	−0.400	−0.401	—	0.001
C	16	−0.018	−0.018	—	—
C	17	−0.207	−0.207	—	—
C	18	−0.252	−0.253	—	0.001
C	19	−0.240	−0.242	—	0.002
C	20	−0.226	−0.228	—	0.002
C	21	−0.371	−0.367	—	−0.004
H	22	0.251	0.251	—	—
H	23	0.223	0.220	—	0.003
H	24	0.233	0.231	—	0.002
H	25	0.262	0.261	—	0.001
H	26	0.267	0.262	—	0.005
H	27	0.264	0.263	—	0.001
H	28	0.266	0.265	—	0.001
H	29	0.265	0.265	—	—
H	30	0.272	0.271	—	0.001
H	31	0.272	0.271	—	0.001
H	32	0.274	0.273	—	0.001
H	33	0.261	0.260	—	0.001
H	34	0.264	0.263	—	0.001
H	35	0.242	0.239	—	0.003
H	36	0.234	0.228	—	0.006
H	37	0.231	0.229	—	0.002
Cl	38	−0.036	—	−0.024	−0.012
Cl	39	−0.025	—	−0.036	0.011
N	40	**−0.697**	**—**	**−0.682**	**−0.015**
N	41	**−0.670**	**—**	**−0.652**	**−0.018**
N	42	−0.576	—	−0.567	−0.009
C	43	−0.287	—	−0.286	−0.001
C	44	−0.314	—	−0.316	0.002
C	45	0.591	—	0.589	0.002
C	46	0.143	—	0.143	—
C	47	−0.044	—	−0.041	−0.003
C	48	−0.038	—	−0.048	0.010
C	49	−0.249	—	−0.247	−0.002
C	50	−0.249	—	−0.246	−0.003
C	51	−0.232	—	−0.234	0.002
H	52	0.245	—	0.240	0.005
H	53	0.232	—	0.238	−0.006
H	54	0.241	—	0.239	0.002
H	55	0.234	—	0.239	−0.005
H	56	0.416	—	0.421	−0.005
H	57	0.442	—	0.441	0.001
H	58	0.278	—	0.278	—
H	59	0.277	—	0.279	−0.002
H	60	0.270	—	0.271	−0.001

a Reference for atoms is shown in Fig. 4.

Table 4 presents the occupancy percentages of the bonds that were more affected after the clopidogrel–clonidine interaction. The presence of sigma and pi bonds between the O4 and C14 of clopidogrel can be observed. The highest variation in the occupancy percentage, when the molecules interact, occurs in the π bond of O4. For isolated clopidogrel, there is 65.82% occupancy in the O4 p orbital and 34.18% in the C14 p orbital. When the molecules interact, the occupancy percentage of O4 increases to 67.52%, while that of C14 decreases to 32.48%, although its occupancies are redistributed by 0.24% and 0.26%, respectively, on the s orbital. This may be due to the fact that the proximity of H56 and H57 of clonidine distorts the double bond, increasing its occupancy. In turn, the H56 and H57 atoms (Table 4) decrease their occupancy percentage in the original bond after the H-bond formation. Similar findings are found for the H36 and H37 atoms, reducing their participation in the original C–H bond after the H-bond formation (Table 4).

Occupancy percentage of the bonds that participate in the interactions

**Table:** 

Clopidogrel	Before interaction	After interaction
O4=C14	BD (1) O 4–C 14	BD (1) O 4–C 14
	(66.24%) O 4 s (40.80%) p (59.20%)	(66.66%) O 4 s (40.85%) p (59.15%)
	(33.76%) C 14 s (33.21%) p (66.79%)	(33.34%) C 14 s (32.69%) p (67.31%)
	BD (2) O 4–C 14	BD (2) O 4–C 14
	(65.82%) O 4 s (0.00%) p (100.00%)	(67.52%) O 4 s (0.24%) p (99.76%)
	(34.18%) C 14 s (0.00%) p (100.00%)	(32.48%) C 14 s (0.26%) p (99.74%)
C21–H36	BD (1) C 21–H 36	BD (1) C 21–H 36
	(61.75%) C 21 s (27.16%) p (72.84%)	(61.81%) C 21 s (27.16%) p (72.84%)
	(38.25%) H 36 s (100.00%)	(38.19%) H 36 s (100.00%)
C21–H37	BD (1) C 21–H 37	BD (1) C 21–H 37
	(61.73%) C 21 s (27.26%) p (72.74%)	(61.88%) C 21 s (27.27%) p (72.73%)
	(38.27%) H 37 s (100.00%)	(38.12%) H 37 s (100.00%)

**Table:** 

Clonidine	Before interaction	After interaction
N40–H56	BD (1) N 40–H 56	BD (1) N 40–H 56
	(71.71%) N 40 s (30.54%) p (69.46%)	(72.87%) N 40 s (32.46%) p (67.54%)
	(28.29%) H 56 s (100.00%)	(27.13%) H 56 s (100.00%)
N41–H57	BD (1) N 41–H 57	BD (1) N 41–H 57
	(72.11%) N 41 s (26.46%) p (73.54%)	(72.13%) N 41 s (26.52%) p (73.48%)
	(27.89%) H 57 s (100.00%)	(27.87%) H 57 s (100.00%)

The interaction of electron delocalization can be reflected quantitatively using stabilization energy (*E*^(2)^), which is estimated based on the theory of second order perturbation.^57^ In the NBO analysis on hydrogen bond systems, the most significant is the charge transfer between lone pairs (LP(Y)) from the Y-atom and the anti-bonding orbitals (BD* (X–H)) of the X–H bonds, which is correlated with the X–H⋯Y interaction intensity and may give a qualitative description of its contribution to the total energy. The charge transfer between the lone pair of an acceptor to the anti-bonding orbital of the donor provides substantial stabilization of the hydrogen bonds. Accordingly, the stabilization energy (*E*^(2)^) is calculated to characterize the hydrogen bond interaction (see Table 5). The donation of electron lone-pairs shows the existence of hydrogen bonds between clopidogrel and clonidine molecules. In general, the strength of the hydrogen bond depends on the electronegativity of the atoms. It can be classified as very strong (*e.g.*, [F⋯H⋯F]^−^), strong (*e.g.*, O–H⋯O=C), or weak (*e.g.*, C–H⋯O) depending on the bond energy, which ranges from 1.7 eV to <0.1 eV, respectively.^50^Table 5 shows the H-bonding energies that are in accordance with the total energy of the C2 system (see Table 2). The interaction LP (1) O4 → BD*(1) N41–H57 is found to be the strongest one with a stabilizing energy of 0.47 eV. From these observations, it can be deduced that the hydrogen bond formed between the O4 of clopidogrel and the H57 of clonidine is the strongest interaction between both molecules, which agrees with the smallest distance obtained for the H57–O4 bonding (see Table 5).

**Table 5:** Second-order perturbation stabilization energies of the H-bonds

Donor NBO (i)	Acceptor NBO (j)	*E* ^(2)^ (eV)	Bond	Distance (Å)
LP (1) O4	BD*(1) N40–H56	0.07	H56–O4	2.21
LP (1) O4	BD*(1) N41–H57	0.47	H57–O4	1.87
LP (1) N40	BD*(1) C21–H36	0.12	H36–N40	2.45
LP (1) N41	BD*(1) C21–H37	0.01	H37–N41	3.00

**Fig. 7:**
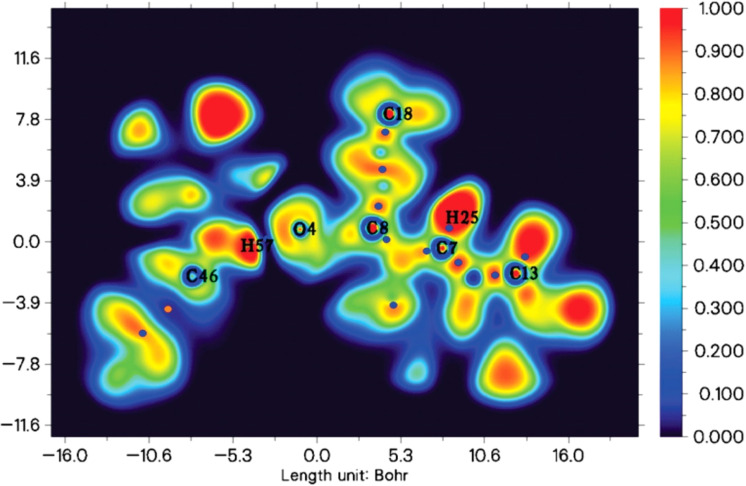
Electron localization function (ELF) map for clopidogrel–clonidine configuration (C2).

The Electron Localization Function (ELF) map from the Multiwfn program,^58^ provides a color-coded representation of the electron distribution of the clopidogrel–clonidine system (Fig. 7). High ELF values, typically shown in warm colors such as red or orange, indicate regions with strongly localized electrons, such as covalent bonds or lone pairs; while low ELF values, often represented in cooler colors like blue, reflect delocalized or non-covalent forces. This visualization aids in understanding the bonding characteristics and electronic interactions. The molecular interaction between clonidine and clopidogrel focuses particularly on the H57 (clonidine) and O4 (clopidogrel) atoms, where this interaction involves non-covalent forces such as hydrogen bonds, according to previous analysis. In the ELF visualization, dark blue regions between atoms highlight voids with low electron localization, suggesting regions dominated by electron delocalization, reinforcing the argument for a weak interaction between clonidine and clopidogrel.

## 4. Conclusions

Since clopidogrel and clonidine medications are typically consumed in combination, it is crucial to examine their molecular interactions. Theoretical parameters serve to interpret the drug properties and predict the bonding in their chemical environment. In general, the frontier orbital energies indicate that clonidine has greater sensitivity to an electrophilic attack, while clopidogrel presents more tendency to donate electrons. The bandgap energy indicates that the neutral clonidine molecule is slightly more reactive than clopidogrel. This result predicts a physical interaction between the molecules. The NBO analysis corroborates this finding. In general, the chemical potential is similar for all the species. This parameter indicates that the electron density can exchange, but the direction is not remarkable and easy to predict. The chemical hardness and softness parameters are also very similar for both molecules. Therefore, clopidogrel and clonidine drugs have similar reactivity in water. Clonidine presents higher electrophilicity values than clopidogrel, indicative that it is more resistant to changes in its electronic distribution.

The MEP map shows that negative charge density is concentrated on the oxygens (clopidogrel), while the positive charge density is located on the nitrogens (clonidine). The TDOS plot shows that the clopidogrel–clonidine system is more stable than the isolated molecules. New states appear in the zone close to the Fermi level after interactions; however, the size of the remanent gap plus the absence of states exactly at the Fermi level coincides with the physical interactions between the drugs. The lowest energy interaction is attained by the formation of four H-bonds (1.87–3.00 Å). The intensive peak at 3360 cm^−1^ in the IR spectrum could be associated with the formed H-bonds. The spectra also confirm the absence of degradation or chemical bonds between the drugs.

## Data availability

Data for this article are available at Repositorio Institucional CONICET Digital at https://ri.conicet.gov.ar/.

## Conflicts of interest

There are no conflicts of interest to declare.
